# Evaluating Wearable Devices for Remote Monitoring in Psychosis: Pilot Study Nested Within the CONNECT Cohort Study

**DOI:** 10.2196/86049

**Published:** 2026-07-07

**Authors:** Siân Bladon, John Ainsworth, Roberto Cahuantzi, Matteo Cella, Richard J Drake, Emily Eisner, Richard Emsley, Sophie Faulkner, Kathryn Greenwood, Andrew Gumley, Gillian Haddock, Kimberley Kendall, Alex Kenny, Jane Lees, Shôn Lewis, Glen P Martin, Matthias Schwannauer, Matthew Sperrin, James T R Walters, Annabel E L Walsh, Pauline Whelan, Til Wykes, Sandra Bucci

**Affiliations:** 1 Division of Informatics, Imaging and Data Sciences, School of Health Sciences, University of Manchester, Vaughan House, Manchester, United Kingdom; 2 Division of Psychology and Mental Health, School of Health Sciences, University of Manchester, Jean McFarlane Building, Oxford Road, Manchester, M13 9PY, United Kingdom, 441613066000, ext 60422; 3 Department of Psychology, Institute of Psychiatry, Psychology and Neuroscience, King's College London, London, United Kingdom; 4 South London and Maudsley NHS Foundation Trust, London, United Kingdom; 5 Greater Manchester Mental Health NHS Foundation Trust, Manchester, United Kingdom; 6 Department of Biostatistics and Health Informatics, Institute of Psychiatry, Psychology and Neuroscience, King's College London, London, United Kingdom; 7 School of Psychology, University of Sussex, Falmer, United Kingdom; 8 Research and Development Department, Sussex Partnership NHS Foundation Trust, Hove, United Kingdom; 9 School of Health and Wellbeing, University of Glasgow, Glasgow, United Kingdom; 10 NHS Greater Glasgow and Clyde, Glasgow, United Kingdom; 11 Centre for Neuropsychiatric Genetics and Genomics, Cardiff University, Cardiff, United Kingdom; 12 McPin Foundation, London, United Kingdom; 13 School of Health in Social Science, University of Edinburgh, Edinburgh, United Kingdom

**Keywords:** psychosis, schizophrenia spectrum disorders, severe mental illness, digital remote monitoring, wearable devices, passive sensing, smartwatch, fitness tracker

## Abstract

**Background:**

Digital remote monitoring technologies, including smartphones and wearables, offer promising avenues for early detection of psychosis relapse. However, selecting devices that are acceptable to participants and produce high-quality data remains challenging.

**Objective:**

The aim of this nested pilot study was to assess the acceptability and data quality of 3 commercially available wearable devices in people with psychosis recruited to the CONNECT cohort study.

**Methods:**

Participants recruited to the CONNECT study before July 31, 2024, were included in the pilot study and selected 1 of 3 wearable devices: a Fitbit Charge 5, Samsung Galaxy Watch 5, or Apple Watch SE. Baseline demographics were compared between device groups. Acceptability of devices to participants was assessed through a Wearable Device Satisfaction Questionnaire after 3 months of use, with the proportion of positive responses to each question calculated and compared. Data completeness was also assessed by calculating the number (and percentage) of valid days of step count, heart rate, and sleep data, and comparing between groups. Data quality was assessed through summarizing the amount of troubleshooting required, additional metrics available from the wearables, and continuity of data completeness by calculating the proportion of participants with at least 3 days of heart rate data per week for the first 20 weeks of follow-up. Predefined criteria were used to determine the next steps for the wider CONNECT study: if one device was superior, this would be selected; if none were found to be superior and the Fitbit was found to be noninferior, then Fitbit would be retained.

**Results:**

Of the first 107 participants recruited to CONNECT, 105 were included in the pilot study evaluation. The Samsung Galaxy Watch was selected most frequently by participants (46/105, 43.8%), followed by the Apple Watch (27/105, 25.7%), and Fitbit Charge (23/105, 21.9%). Differences in participant demographics were observed across device groups. Self-reported acceptability after use did not differ substantially between devices. However, in terms of data completeness, the median proportion of valid heart rate data days was significantly lower for Samsung Galaxy (median 31.2%, IQR 8.5%-46.0%) compared to Fitbit (median 80.1%, IQR 26.7%-95.0%; *P*=.003) and Apple Watch (median 49.3%, IQR 21.5%-86.0%; *P*=.02). There was no significant difference between Fitbit and Apple Watch. Similar patterns were observed for step count and sleep data. The Samsung Galaxy Watch required more frequent troubleshooting for data flow issues and lacked additional physiological metrics, available from the other devices.

**Conclusions:**

Due to comparatively lower data quality and technical performance, the Samsung Galaxy Watch was discontinued for use in the subsequent phase of the CONNECT study. The study highlights the importance of incorporating nested evaluations of devices in long-term research.

## Introduction

Schizophrenia spectrum disorders affect approximately 20 million people globally and are associated with substantial clinical, functional, and economic burden [1]. Relapses following a first episode of psychosis are common [2] and are associated with poorer long-term prognosis, more hospitalization, and lower quality of life [3-5]. Digital remote monitoring (DRM) technologies, such as smartphones and wearable devices (eg, smartwatches and fitness trackers), offer the potential to detect real-time changes in physiological and behavioral metrics prior to clinical deterioration and support timely interventions to prevent a psychosis relapse [6,7]. Active symptom monitoring (ASM) via smartphone apps has been found to be both feasible and acceptable in people with psychosis and schizophrenia, with the potential to reduce rates of relapse when these data are incorporated into care pathways [8]. However, a limitation of ASM is the burden it places on the individual to complete questionnaires, sometimes multiple times per day. With the use of smartphones and wearables increasing, this creates new opportunities to collect data in a more passive way, requiring less active input and engagement from the user. Sensors in smartphones and wearable devices (eg, accelerometers or GPS sensors) continuously collect data that can be used to derive features relating to important behaviors such as sleep, physical activity, or mobility [9]. In this context, the CONNECT cohort study [10] was established with the aim of integrating ASM data from a self-monitoring app and passive sensing data from smartphones and wearables into a risk prediction algorithm for estimating short-term relapse risk in individuals with psychosis.

For DRM to be successfully implemented in clinical settings, the usability, acceptability, and data quality of the underlying technologies are crucial [11]. While various commercial and research-grade wearables are available and have been used in studies including people with psychosis and schizophrenia [12-14], choosing which device to use is challenging. Research-grade devices may offer greater accuracy than commercially available wearables when benchmarked against gold-standard methods. However, commercial devices have shown good engagement in longer-term studies [15,16] and been found to be more acceptable to participants [17,18]. A further advantage of commercial wearables is that they can capture multiple behaviors within the same device, with most providing at least physical activity, sleep, and heart rate metrics. However, the evidence on the data quality and accuracy of wearables for these core measures remains mixed. A 2022 systematic review by Germini et al [19] of 65 studies examined the accuracy of different wearable devices for measuring physical activity. The review found that Fitbit devices were more reliable than Apple or Garmin devices for recording step count. However, a larger review by Fuller et al [20] of 158 papers reported that Apple and Samsung Watches showed lower step count error than Fitbit devices under controlled conditions. A 2026 systematic review focused specifically on Apple Watch accuracy included 3 studies assessing step count and found moderate correlations with reference measures [21]. For heart rate, both Germini et al [19] and Fuller et al [20] found that Apple Watches were more accurate than Fitbit devices. The review by Lambe et al [21], which included 38 studies assessing heart rate measurement, found that Apple Watches tend to overestimate resting heart rate and underestimate heart rate during exercise, although agreement with reference standards was higher in more recent models. For sleep measurement, a 2023 study comparing 5 wearable devices against gold-standard polysomnography, including the Galaxy Watch 5, Fitbit Sense 2, and Apple Watch 8, found that the Galaxy Watch and Fitbit devices performed better overall than the Apple Watch [22]. In the review by Lambe et al [21], 3 studies assessed Apple Watch sleep accuracy and reported good performance in distinguishing sleep from wake states, but poorer accuracy for differentiating sleep stages. Similar conclusions have been reported in other reviews and individual comparative studies across multiple wearable brands [23-25]. In addition to variation in wearable models, there was variation in the criterion measure used to estimate measurement error across the included studies. For example, studies assessing heart rate accuracy compared commercial devices against different reference standards, including gold-standard 12-lead electrocardiogram and research-grade chest-strap heart rate monitors. Lambe et al [21] and Fuller et al [20] also highlighted the rapid obsolescence of devices evaluated in the literature. In the review by Fuller et al [20], for example, only 8 of the 47 different devices included were still available by the time of publication. Together, these issues highlight the difficulty of using the published literature to guide device selection, as both hardware and software proprietary algorithms evolve rapidly, and there is no standardized framework for evaluating wearables.

In terms of acceptability and usability of commercial wearables, previous studies have assessed this using either quantitative measures of data completeness [26,27] or qualitative methods such as questionnaires with items on ease of use, design, functionality, and durability of devices [26,28-31]. One study in older adults found no significant difference in usability from the participants’ perspective between a Fitbit Charge 3 and an Apple Watch 3, but significant differences in the amount of data collected [26] after 2 weeks of use. Another study in adults aged 20‐30 years compared 2 Fitbit models with 2 Samsung Galaxy Watch models following 30 days of use and found that the Galaxy Watch 5 received the overall satisfaction scores, with battery life and design identified as important features influencing acceptability [32]. Mannhart et al [28] similarly invited participants to wear 5 different wearables and select a preferred option; the Apple Watch 6 was chosen most often, followed by the Withings ScanWatch, Fitbit Sense, and Samsung Galaxy Watch 3. In that study, device design and prior familiarity with the brand appeared to influence preferences. However, the study was conducted under controlled conditions, and participants wore each device for only 30 seconds. Taken together, these contrasting findings across different populations, alongside the limited evidence on acceptability during long-term use in uncontrolled settings, make it difficult to rely on the existing literature to inform device selection for future studies.

While these prior studies provide some evidence on device accuracy, important uncertainties remain about the suitability of currently available commercial wearables for more longer-term use, particularly in relation to acceptability, feasibility, and data completeness. Therefore, to inform device selection prior to wider deployment across the CONNECT study cohort, we conducted a nested evaluation with all participants recruited up to July 31, 2024. This evaluation compared 3 readily available commercial wearable devices selected for their accessibility and suitability for data collection in routine research settings. Specific objectives were to evaluate which device (1) most participants chose to use, (2) was most acceptable to participants after using it, and (3) provided the highest quality data during the monitoring period.

## Methods

### Ethical Considerations

Ethics approval was obtained from the Health Research Authority and Research Ethics Committee (REC reference: 23/WM/0044). The study was registered at the UK’s Clinical Study Registry (International Standard Randomised Controlled Trial Number 24746936) [33]. Informed consent was obtained via a signed paper consent form, signed electronic consent form, or audio-recorded consent. Participants were able to withdraw their consent at any time during the study, without giving a reason. All research data were pseudoanonymized, and unique study IDs were assigned to participants and used instead of personally identifiable data. Participants received £20 (GBP £1=US $1.34 as of June 15, 2026) reimbursement for their time completing each clinical assessment, reimbursement for travel expenses, and data costs (£10 per month) were covered for the study duration.

### Participant Recruitment and Eligibility Criteria

Full details of the CONNECT cohort study protocol are reported elsewhere [33]. In brief, CONNECT is a prospective, observational cohort study aiming to recruit a total of 1100 individuals with a schizophrenia spectrum diagnosis. Recruitment is carried out through mental health services in National Health Service (NHS) Mental Health Trusts/Health Boards across 6 UK locations (Manchester, Sussex, South London, Glasgow, Edinburgh, and South Wales) from March 2024. Clinical staff at mental health services identify potential participants and provide them with initial information about the study, before gaining consent to pass on contact details to study researchers. Researchers then contact individuals to confirm eligibility for the study, provide further information, and obtain formal consent for taking part. Eligible individuals are required to meet the following criteria: (1) ≥16 years of age; (2) experienced at least 1 acute episode of psychosis within the past 2 years that led to unscheduled acute care; (3) clinical diagnosis of a schizophrenia spectrum disorder (International Classification of Diseases-10 F20-F29); (4) at the time of recruitment, their presentation did not include severe acute symptoms; and (5) capacity to provide informed consent. Exclusion criteria included individuals who had experienced a relapse within the previous 12 weeks, as confirmed by their treating clinician, or insufficient English language proficiency. All participants continued to receive usual multidisciplinary care. During the consent process, researchers explain that participation in the study is voluntary, they can withdraw consent at any time, and withdrawing will not impact access to usual care.

For this nested pilot study, we invited the first 100 participants enrolled in the CONNECT cohort between March 14, 2024, and July 31, 2024, to take part, with a minimum of 10 participants at each of the 6 recruiting sites. No formal sample size calculation was performed, with the sample size instead determined by anticipated recruitment rates in the first few months of the study and the need to be able to make a timely decision about devices for the remainder of the study.

### Study Procedure

At enrollment, participants were offered 1 of 3 commercial-grade wrist-worn wearable devices for the study period: a Fitbit Charge 5, a Samsung Galaxy Watch 5, or an Apple Watch SE. All 3 devices record step count, heart rate, physical activity, and sleep data. We selected commercially available wearables rather than research-grade devices because previous similar studies demonstrated good uptake and adherence with commercial-grade devices during longitudinal monitoring [15,16]. Device selection was also informed by cost, the range of available sensors, and the feasibility of accessing data for research purposes, including higher-resolution data from Fitbit intraday data and Apple SensorKit. In addition, we considered the extent to which each device could be integrated with the relevant smartphone operating system, either through direct operating system functionality or via an application programming interface. iPhone users could select either an Apple Watch or a Fitbit, while Android users could choose an Android smartwatch or a Fitbit. Those who already owned a compatible device could continue using it, and those without a suitable smartphone were provided with an Android smartphone. All participants received an initial onboarding session following their baseline assessment, where a researcher helped set up the wearable. After onboarding, participants began using the device, and passive data collection commenced. Participants were encouraged to continue their daily activities as usual while using the study devices and to respond to app alerts and notifications when prompted. All participants were provided with an App and Wearable Guide containing practical instructions, frequently asked questions, and guidance on the use of the app, wearable device, and study phone. All participants were invited to complete follow-up assessments between 3 and 6 months after enrollment.

### Data Collection

Baseline data were collected on demographics, socioeconomic factors, medical history, and device information. At follow-up assessments, participants completed a Wearable Device Satisfaction Questionnaire (WDSQ), comprising 29 items rated on a 7-point scale. Demographics and self-report data were maintained in REDCap (Research Electronic Data Capture); Vanderbilt University) [34]. A data monitoring log tracked data collection and troubleshooting activities related to smartphones and wearable devices; issues were initially addressed locally and escalated to members of our software team when needed. Researchers at each site provided information on the length of the onboarding sessions. Passive data from smartphones and wearable sensors were collected via the CONNECT platform. The CONNECT mobile app (the participant-facing component of the CONNECT platform) also collects ASM data through daily questionnaires. The CONNECT platform was developed and managed by the Digital Health Software Team at the University of Manchester [35], with modified versions of the CareLoop [36] and Remote Assessment of Disease and Relapses (RADAR-base) [37] technologies providing the underlying digital infrastructure to support it and outsourced support from Hyve. Data were stored on UK-based Amazon Web Services cloud servers, with secure transfer to the University of Manchester’s Research Data Storage system for analysis.

### Patient and Public Involvement

This pilot study was developed with strong involvement from the CONNECT Lived Experience Advisory Panel, a diverse group of 12 individuals recruited from across the 6 study sites, supported by the McPin Foundation. The Lived Experience Advisory Panel contributed to the design and implementation of the pilot study, helped interpret the findings, and advised on how the results should inform decisions in the main CONNECT cohort study.

### Data Analysis

#### Participant Exclusions and Device Grouping

Participants who withdrew from the study during follow-up and withdrew consent to use data already collected were excluded from all analyses. Participants who did not consent to use a wearable or who were using their own compatible device were included in the analysis for objective 1 but then excluded from all further analyses because prior familiarity with the device may have influenced both satisfaction ratings and data completeness. Participants with no passive data available were excluded from the quantitative analysis in objectives 2 and 3. For some, this was due to issues with the downloading and merging of data files, which affected participants in each wearable group. For others (all in the Samsung group), the lack of data appeared to be due to participants either withdrawing or disengaging with the study soon after onboarding.

For objective 1, participants were grouped by the device they selected to use at baseline. For summary analyses of the WDSQ, participants who changed devices during the follow-up period were categorized according to the device they were using at the time of the follow-up assessment. For the quantitative analyses, participants who switched devices contributed 2 observation periods, one for each device, and both periods were included in the analyses.

#### Objective 1: Which Wearable Device Do Most Participants Choose to Use?

The number of participants selecting each wearable device at onboarding was calculated, and baseline demographics were summarized by device and compared using either a Kruskal-Wallis test with Bonferroni post hoc test or a chi-square test. Continuous variables were summarized as mean and SD (or median and IQR), and discrete variables reported as the number and percentage of participants.

#### Objective 2: Which Device Is Most Acceptable to Participants After Using It?

Participants’ responses to each item of the WDSQ were summarized. The items were grouped into 7 categories: ease of use, functionality and practicality, satisfaction, device app, physical activity behavior, smartphone, and other. The number of participants responding positively to each item was compared between device groups. A positive response was defined as either agree and strongly agree or disagree and strongly disagree, depending on the question’s valence. To summarize across the items, the devices were ranked for each question by the proportion of positive responses, and the number of times each device was ranked in each position was calculated. To assess data completeness, the number of valid days where any sleep, heart rate, and step count data had been recorded was calculated for each participant. These variables were selected as they are recorded by all 3 devices. Data completeness was used as a proxy measure of acceptability on the assumption that users are more likely to wear, and therefore record more data with, a device they find acceptable. For each participant, the total number of possible data collection days was calculated as the number of days between their onboarding date and either the pilot study end date (October 21, 2024) or their date of withdrawal, whichever came first. The number of actual days with data was then expressed as a percentage of this total. The median and IQR were calculated for each wearable group and compared using a Kruskal-Wallis test with a Bonferroni post-hoc test, with a *P* value <.05 considered significant.

#### Objective 3: Which Device Provides the Highest Quality Data During the Monitoring Period?

To assess data completeness at a more granular level, the number of valid heart rate hours, defined as hours with at least 1 recorded observation, was calculated as a proportion of the total possible hours for each participant, assuming continuous 24-hour wear for all possible days of follow-up. Heart rate was used, as this is recorded at a similar frequency by all 3 devices and is recorded regardless of whether participants are sleeping or moving. The median and IQR were calculated for each wearable group and compared using a Kruskal-Wallis test with a Bonferroni post-hoc test, with a *P* value <.05 considered significant. To assess the continuity of data completeness, we calculated the proportion of participants in each device group who had at least 3 days of valid heart rate data per week, for each of the first 20 weeks of follow-up. Week 20 was used as the cutoff point due to the reduced number of participants remaining in each group beyond that time. The amount of troubleshooting required, the estimated onboarding times, and the additional metrics available were summarized for each device. To assess the correlation between passive data completeness and completed ASM questionnaires, the proportion of participants in each group who had completed at least 3 questionnaires per week was calculated and plotted over time. To assess whether differences in passive data completeness were attributable to differences in the wearable groups, a Poisson model was developed with the number of days of heart rate data as the outcome, wearable choice as the independent variable, and the log number of completed ASM questionnaires as an offset (as a proxy for overall study engagement). Two additional models adjusted for baseline demographics, including age, ethnic group, sex, and deprivation quintile, with an offset of either the log number of ASM encounters or the log number of possible days of data.

### Guiding Criteria for Device Evaluation and Selection

Following the initial 6-month evaluation period, we used a set of predefined decision-making criteria outlined in our statistical analysis plan to determine the next steps. First, if one device clearly outperformed the others in terms of both data quality and participant acceptability, it would be recommended for use across the remainder of the CONNECT cohort study. If no significant differences in performance were observed, a noninferiority analysis would be conducted to assess whether the Fitbit was noninferior to the smartwatches. Should Fitbit meet the criteria for noninferiority, it would be selected as the preferred device due to its lower cost and ease of use. However, if the findings were inconclusive, data collection would continue for an additional 6 months, and the analyses would be repeated.

## Results

### Characteristics of the Sample

**Figure 1.**
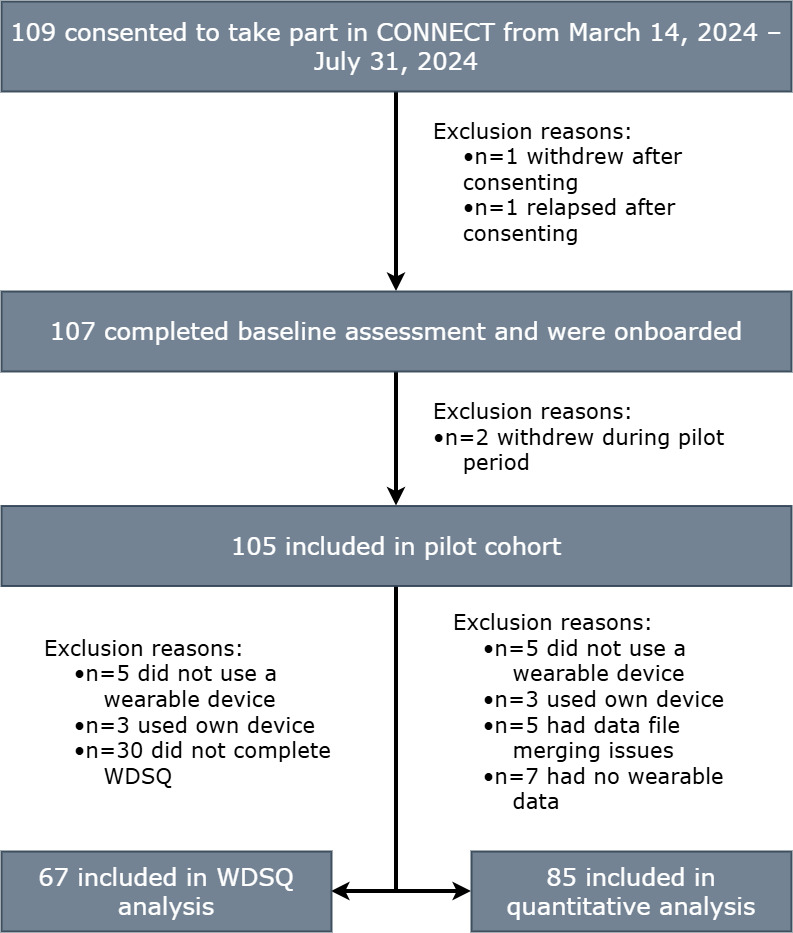
Flow of participants through the CONNECT pilot study. The first 105 participants recruited to the CONNECT cohort study were included in the pilot study. WDSQ: Wearable Device Satisfaction Questionnaire.

A total of 107 participants were recruited between March 14, 2024, and July 31, 2024. While the study protocol specified a minimum of 10 participants per recruitment site, this was not met at 2 recruitment sites, which each enrolled 7 participants. As the overall sample exceeded the target of 100 participants, a decision was made to proceed with the analyses. After exclusions, a final sample of 105 participants was included in the pilot cohort (Figure 1). The median age of participants was 33.0 (IQR 24.0-45.0) years, with 56.2% male, 53.3% identified as White, and 39% lived in areas falling within the most deprived quintile according to the Index of Multiple Deprivation (IMD; Table 1).

**Table 1.** Baseline characteristics of the 105 participants included in the CONNECT pilot study.

Demographic characteristic	All (n=105)	No wearable (n=6)	Apple Watch (n=28)	Fitbit (n=24)	Samsung Galaxy (n=47)	*P* value
Age (years), median (IQR)	33.0 (24.0-45.0)	38.5 (34.0-43.8)	26.0 (21.0-35.0)	35.5 (23.8-54.3)	34.0 (26.5-45.5)	.04
Age group (years), n (%)						.27
16‐20	7 (6.7)	0 (0)	4 (14.3)	2 (8.3)	1 (2.1)	
21‐30	38 (36.2)	1 (16.7)	12 (42.9)	6 (25)	19 (40.4)	
31‐40	23 (21.9)	2 (33.3)	7 (25)	5 (20.8)	9 (19.1)	
41‐50	18 (17.1)	2 (33.3)	4 (14.3)	4 (16.7)	8 (17)	
51‐60	13 (12.4)	1 (16.7)	1 (3.6)	3 (12.5)	8 (17)	
61‐70	6 (5.7)	0 (0)	0 (0)	4 (16.7)	2 (4.3)	
Ethnic group, n (%)						.40
Asian or Asian British	13 (12.4)	1 (16.7)	4 (14.3)	2 (8.3)	6 (12.8)	
Black or Black British or Caribbean or African	22 (21)	1 (16.7)	4 (14.3)	3 (12.5)	14 (29.8)	
White	56 (53.3)	3 (50)	17 (60.7)	15 (62.5)	21 (44.7)	
Mixed or multiple groups	10 (9.5)	0 (0)	2 (7.1)	2 (8.3)	6 (12.8)	
Other	4 (3.8)	1 (16.7)	1 (3.6)	2 (8.3)	0 (0)	
Sex, n (%)						.87
Female	46 (43.8)	2 (33.3)	14 (50)	10 (41.7)	20 (42.6)	
Male	59 (56.2)	4 (66.7)	14 (50)	14 (58.3)	27 (57.4)	
Employment status^a^, n (%)						
Self-employed	1 (1)	0 (0)	0 (0)	1 (4.2)	0 (0)	.27
Employed	16 (15.2)	0 (0)	7 (25)	4 (16.7)	5 (10.6)	.32
Out of work and looking for work	20 (19)	2 (33.3)	4 (14.3)	3 (12.5)	11 (23.4)	.45
Out of work and not looking	20 (19)	1 (16.7)	4 (14.3)	4 (16.7)	11 (23.4)	.85
Unable to work	25 (23.8)	2 (33.3)	2 (7.1)	7 (29.2)	14 (29.8)	.08
Voluntary work	7 (6.7)	1 (16.7)	2 (7.1)	2 (8.3)	2 (4.3)	.47
Student	13 (12.4)	0 (0)	9 (32.1)	2 (8.3)	2 (4.3)	.005
Retired	4 (3.8)	0 (0)	0 (0)	2 (8.3)	2 (4.3)	.42
IMD^b^ quintile, n (%)						.02
1	41 (39)	1 (16.7)	8 (28.6)	6 (25)	26 (55.3)	
2	26 (24.8)	2 (33.3)	6 (21.4)	6 (25)	12 (25.5)	
3	18 (17.1)	1 (16.7)	6 (21.4)	5 (20.8)	6 (12.8)	
4	13 (12.4)	1 (16.7)	4 (14.3)	6 (25)	2 (4.3)	
5	6 (5.7)	1 (16.7)	4 (14.3)	1 (4.2)	0 (0)	
Missing	1 (1)	0 (0)	0 (0)	0 (0)	1 (2.1)	
In receipt of welfare benefits, n (%)						.04
Yes	87 (82.9)	5 (83.3)	19 (67.9)	22 (91.7)	41 (87.2)	
No	16 (15.2)	1 (16.7)	9 (31)	1 (4.2)	5 (10.6)	
Prefer not to say	1 (1)	0 (0)	0 (0)	1 (4.2)	0 (0)	
Missing	1 (1)	0 (0)	0 (0)	0 (0)	1 (2.1)	

a Participants could select more than 1 option.

b IMD: Index of Multiple Deprivation.

### Objective 1: Which Wearable Device Do Most Participants Choose to Use?

At onboarding, 43.8% (46/105) of participants selected the Samsung Galaxy Watch, 25.7% (27/105) chose the Apple Watch, and 21.9% (23/105) chose the Fitbit device. An additional 3 participants used a device they already owned, one from each device group. In total, 6 of 105 (5.7%) participants declined to use a wearable at onboarding. Over the study duration, 5 participants changed their device: 2 switched to a different device, 2 discontinued wearable use, and 1 participant who had initially declined a wearable began using one.

Baseline demographics by initial device group are displayed in Table 1 (Table S1 in Multimedia Appendix 1 gives demographics by site). Notable demographic differences were observed across the device groups. Apple Watch users were younger and included a greater proportion of students, and the lowest proportion of participants receiving welfare benefits. The Fitbit group had the highest proportion of participants in the 61‐ to 70-year age group (4/24, 16.7%) and the highest rate of welfare benefit receipt (22/24, 91.7%). The Samsung Galaxy Watch group included the highest proportion of Black or Black British or Caribbean or African participants (14/47, 29.8%), the largest percentage of individuals residing in the most deprived IMD quintile (26/47, 55.3%), and the lowest proportion of employed participants (5/47, 10.6%).

### Objective 2: Which Device Is Most Acceptable to Participants After Using It?

A total of 76 (72.4%) participants completed a follow-up assessment during the study period, and 70 (66.7%) had completed at least 1 item on the WDSQ. The distribution of positive responses to each WDSQ item is presented in Table 2. Overall, no device consistently scored more positively than the others, and variability was observed even within similar question categories. For example, within the “ease of use” domain, Samsung Galaxy Watch users had the highest proportion of positive responses to the item “The device is easy to use” (22/25, 88%), compared with Fitbit (12/17, 70.6%) and Apple Watch (16/25, 64%) users. However, responses to “I learned to use the device quickly” were similar across all groups (Apple Watch and Samsung Galaxy: 72%(18/25); Fitbit: 70.6% (12/17)). When responses were ranked by the percentage of positive endorsements for each question, the Apple Watch and Fitbit were each ranked first or joint first for 11 items, while the Samsung Galaxy Watch was ranked first or joint first for 9 items.

**Table 2.** Summary of responses to each item on the Wearable Device Satisfaction Questionnaire (WDSQ)^a^.

Topic and question	Positive responses		
	Apple Watch (n=25), n (%)	Fitbit (n=17), n (%)	Samsung Galaxy (n=25), n (%)
Ease of use			
I easily remember how to use my device	19 (76)	15 (88.2)	20 (80)
I can easily sync my device with the smartphone	22 (88)	12 (70.6)	19 (76)
I can easily wear my device each day	17 (68)	14 (82.4)	20 (80)
I learned to use the device quickly	18 (72)	12 (70.6)	18 (72)
I found that I lost or misplaced or damaged the device easily	16 (64)	12 (70.6)	12 (48)
I think that I would need more training or support to use the device in the future	19 (76)	13 (76.5)	13 (52)
The device fit into my daily routine easily	19 (76)	14 (82.4)	19 (76)
I easily remember how to sync my device with the smartphone	20 (80)	10 (58.8)	18 (72)
The device is easy to use	16 (64)	12 (70.6)	22 (88)
Functionality and practicality			
The device broke down frequently	21 (84)	9 (52.9)	19 (76)
The device is a good size	22 (88)	15 (88.2)	20 (80)
The device is useful	21 (84)	14 (82.4)	20 (80)
The device does everything I would expect it to do	18 (72)	15 (88.2)	23 (92)
The device meets my needs	21 (84)	13 (76.5)	20 (80)
Satisfaction			
I would like to continue using the device in the future	21 (84)	15 (88.2)	22 (88)
The device is fun to use	18 (72)	15 (88.2)	21 (84)
The device works the way I want it to work	21 (84)	13 (76.5)	21 (84)
I would recommend the device to a friend	18 (72)	15 (88.2)	22 (88)
I am satisfied with the device	20 (80)	13 (76.5)	21 (84)
Device app			
The device app on the smartphone is easy to use	16 (64)	13 (76.5)	22 (88)
The device app on the smartphone does everything I would expect it to do	19 (76)	13 (76.5)	20 (80)
I learned to use the device app on the smartphone quickly	20 (80)	11 (64.7)	19 (76)
The device app on the smartphone is useful	21 (84)	12 (70.6)	18 (72)
Physical activity behavior			
The device gives me more control over getting physical activity in my daily routine	17 (68)	13 (76.5)	19 (76)
The device helps me keep track of my physical activity	22 (88)	13 (76.5)	21 (84)
The device helps me to be more active	15 (60)	11 (64.7)	18 (72)
Smartphone			
The smartphone is easy to use	20 (80)	14 (82.4)	20 (80)
I learned to use the smartphone quickly	20 (80)	13 (76.5)	18 (72)
Other			
I feel I need to use a device	9 (36)	9 (52.9)	17 (68)

a Number (and percentage) of people responding positively was calculated for each question. In total, 70 (66.7) participants had completed at least 1 item of the WDSQ. A total of 3 were excluded as they were using their own devices. The 5 participants who changed wearables during the pilot follow-up period were included in the group for the device they were using when they completed the WDSQ.

**Figure 2.**
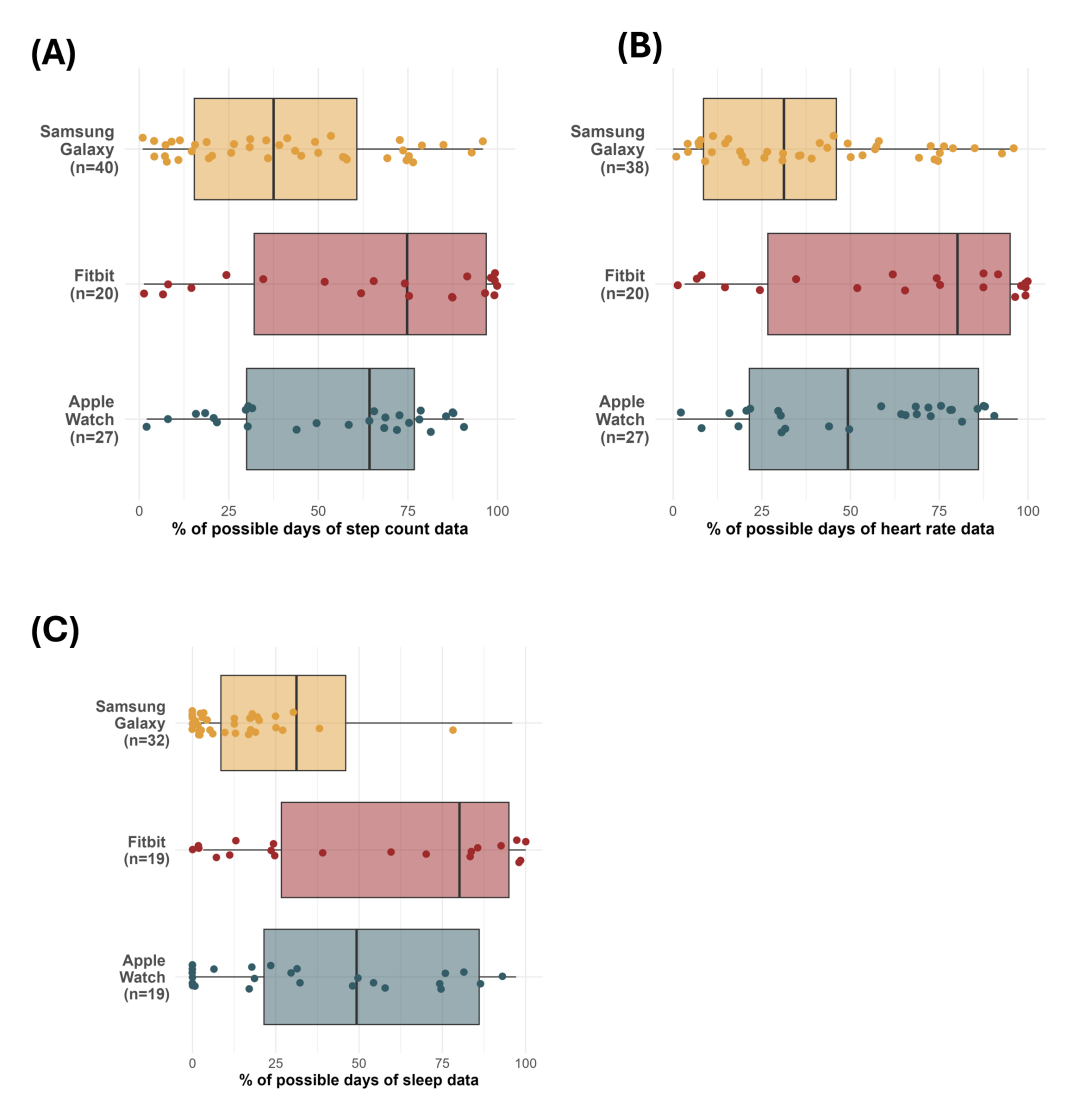
Percentage of possible days with data for (A) step count, (B) heart rate, and (C) sleep data. The total number of participants in each group was 40 for Samsung Galaxy, 20 for Fitbit, and 27 for Apple Watch. Participants were excluded if they did not have any amount of each data type available. For step count, the Samsung Galaxy group had a significantly lower median proportion of valid step count days compared to Fitbit users (*P*=.04) but not Apple Watch users (*P*=.23). For heart rate data, the Samsung Galaxy group was significantly lower than Fitbit (*P*=.003) and Apple Watch (*P*=.02). For sleep data, Samsung Galaxy was significantly lower than Fitbit (*P*<.001) but not Apple Watch (*P*=.16).

After exclusions, there were 85 participants included in the quantitative analysis, of which 2 switched devices during the study period, resulting in 87 analyzable participant-device periods. Step count data were available for all participants, while heart rate data were missing for 2 participants in the Samsung Galaxy group. Sleep data completeness varied by device: 95% (19/20) of Fitbit users had sleep data recorded, compared with 80% (32/40) of Samsung Galaxy users and 70.4% (19/27) of Apple Watch users (Figure 2 and Table S2 in Multimedia Appendix 1). There were no significant differences in the number of possible data collection days across device groups (Table S2 in Multimedia Appendix 1). However, data completeness differed significantly between devices. The Samsung Galaxy group had a lower median proportion of valid step count days (Figure 2A; median 37.5%, IQR 15.4%-60.7%), compared to Fitbit users (median 74.8%, IQR 32.1%-96.9%; *P*=.04) but not Apple Watch users (median 64.3%, IQR 30.0%-76.8%; *P*=.23). For heart rate data (Figure 2B), the Samsung Galaxy group again showed lower data completeness (median 31.2%, IQR 8.5%-46.0%) compared with both Fitbit (median 80.1%, IQR 26.7%-95.0%; *P*=.003) and Apple Watch (median 49.3%, IQR 21.5%-86.0%; *P*=.02). Sleep data (Figure 2C) were also lower for Samsung Galaxy users (median 4.8%, IQR 1.9%-17.6%) than Fitbit (median 49.4%, IQR 12.5%-87.3%; *P*<.001) and lower, though not significantly, than Apple Watch users (median 23.6%, IQR 0%-56.1; *P*=.16).

### Objective 3: Which Device Provides the Highest Quality Data During the Monitoring Period?

When heart rate data were examined at the hourly level, the Fitbit group again showed the highest data completeness, with a median of 53.0% (IQR 21.3%-92.4%) of possible hours (Table S2 and Figure S1 in Multimedia Appendix 1). This was significantly greater than the Samsung Galaxy group (median 15.8%, IQR 3.4%-30.1%; *P*=.001), but not significantly different from the Apple Watch group (median 28.8%, IQR 10.5%-57.6%; *P*=.13).

**Figure 3.**
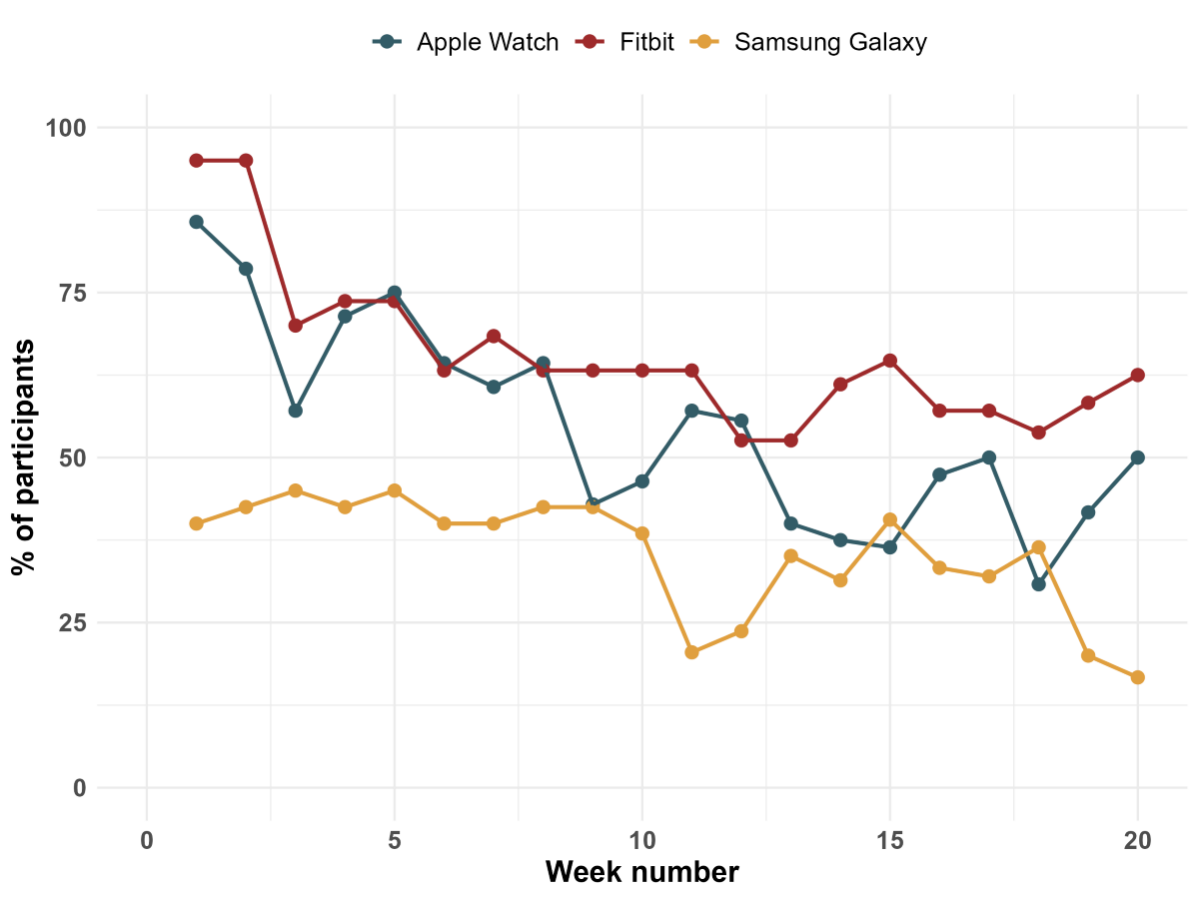
Percentage of participants in each wearable group with at least 3 days of valid heart rate data per week. A day was considered valid if at least 1 heart rate measurement was recorded at any time throughout the day. Heart rate was chosen, as this is the only common continuously measured metric across all 3 devices.

In week 1 of the study, 95% (19/20) of Fitbit users had at least 3 valid days of heart rate data, compared with 85.2% (23/27) of Apple Watch users and only 40% (16/40) of Samsung Galaxy users (Figure 3). By week 20, only 16.7% (3/18) of Samsung Galaxy users continued to meet the 3-day threshold, compared with 57.1% (4/7) of Apple Watch users and 62.5% (5/8) of Fitbit users. Notably, the proportion of Fitbit users with at least 3 valid days of heart rate data per week remained above 50% throughout the 20-week period, whereas the Samsung Galaxy group never exceeded 50% in any week. Correlation of passive data completeness with engagement with the ASM questionnaires was seen across all 3 device groups (Figure S2 in Multimedia Appendix 1). Poisson modeling adjusting for the number of completed ASM encounters showed weak evidence that differences in passive data completeness were due to device choice (Table S6 in Multimedia Appendix 1). Models adjusting for baseline demographics (Table S7 in Multimedia Appendix 1) showed a significant association between being male and wearable data completeness (model ii: incidence rate ratio 1.38, 95% CI 1.01‐1.90), but no significant association between age, ethnic group, employment status, being in receipt of welfare benefits, or deprivation quintile, and wearable data completeness.

Of the 30 troubleshooting queries escalated to the study software team, the majority (n=20, 66.7%) were related to the Samsung Galaxy Watch. In total, 6 (20%) queries concerned the Fitbit, and 4 (13.3%) the Apple Watch. After investigation, 17 of the 20 Samsung Galaxy queries were attributed to a newly enforced privacy policy by Samsung that blocked data transmission. For the Fitbit, 4 of 6 queries were related to hardware malfunction, while the remaining 2 involved data flow problems. Apple Watch issues primarily stemmed from older iOS versions preventing pairing and instances of participants being logged out of the app, which disrupted data upload.

Onboarding time estimates were available for a subset of participants (n=52; Table S3 in Multimedia Appendix 1). Median onboarding time was 60 (IQR 58-98) minutes for the Apple Watch group, 60 (IQR 30-180) minutes for the Samsung Galaxy group, and 90 (IQR 83-120) minutes for the Fitbit group, with no statistically significant difference between them.

Although all 3 devices record a similar range of metrics, there were important differences in the type, resolution, and accessibility of the data (Table S4 in Multimedia Appendix 1). Sleep data and heart rate were recorded at comparable frequencies across devices, but step count data were not: Fitbit records minute-level data, the Apple Watch (via SensorKit) provides data every few seconds, whereas the Samsung Galaxy reports daily totals. In addition, the Fitbit and Apple Watch devices offered further physiological metrics, and the Apple Watch also enabled access to raw accelerometer data via SensorKit.

While sleep data were available for most users across devices, the Samsung Galaxy group had significantly less valid sleep data overall. Furthermore, 98% of the sleep data collected from the Samsung Galaxy Watch was affected by a critical formatting issue, with duplicated start timestamps across multiple sleep stages and missing end timestamps. This error rendered it impossible to derive meaningful sleep metrics, such as sleep duration, from the Samsung Galaxy data.

## Discussion

### Principal Findings

This pilot study aimed to evaluate 3 commercially available wearable devices to determine which device participants were most likely to select, found most acceptable after use, and provided the highest quality data during the study monitoring period. Among the 3 options, the most frequently selected device was the Samsung Galaxy Watch, followed by the Apple Watch and the Fitbit. Despite initial preferences, participant-reported satisfaction was broadly comparable across all devices after use. However, substantial differences emerged in data quality and completeness. Only the Fitbit consistently delivered high volumes of minute-level data and captured a broader range of physiological metrics (Table S4 in Multimedia Appendix 1). Therefore, the Fitbit was retained for use in the larger CONNECT cohort study. The Apple Watch was also retained for 3 key reasons. First, participants selected it more frequently than the Fitbit, and removing a smartwatch option risked reducing study uptake. Second, retaining both devices means we can assess the feasibility of developing a DRM that does not rely on data from a single device. Third, the Apple Watch provides a more comprehensive set of metrics, enhancing feature extraction in a subsample of participants. This enhanced data granularity is particularly valuable for advancing CONNECT’s core objective of developing predictive algorithms to support early identification of relapse risk in psychosis. In contrast, the Samsung Galaxy Watch was discontinued after the initial evaluation due to persistently lower data volumes and quality, limited physiological outputs, and the significant staff time required to troubleshoot data flow issues. While the device was the most preferred choice at baseline and showed similar acceptability to participants after use, retaining it would have introduced inconsistency in data type and quality across participants, with potential consequences for comparability and downstream algorithm development. These discrepancies in findings from the WDSQ and data quality analysis highlight a limitation of using commercial wearables not designed primarily for recording data for research, in that devices may be acceptable to participants, but this does not necessarily result in higher data completeness. The Samsung Galaxy was more frequently chosen by participants from groups that are often under-represented in research, including Black or Black British or Caribbean or African participants, those living in the most deprived IMD quintile, and those not in employment. While the decision taken was data-driven, it may have unintended implications for inclusivity and representativeness in the CONNECT cohort. Additionally, engagement with passive data collection appeared to mirror engagement with ASM data: the Samsung Galaxy group had lower levels of both, suggesting generally reduced engagement with the study among users of this device (Figure S2 in Multimedia Appendix 1).

In our cohort, only 11.4% of participants owned and used a wearable device before joining CONNECT, lower than both UK adult population estimates (41%) [38] and rates among people with psychosis-related disorders (21%) [39]. The Fitbit was least frequently chosen at onboarding, likely reflecting its market positioning as a fitness tracker rather than a fully featured smartwatch. UK survey data show a decline in fitness tracker use alongside rising smartwatch ownership [38]. Similar patterns have been observed internationally, with studies consistently reporting higher Apple Watch uptake compared to Fitbit or Samsung devices. For example, Jia et al [30] found that Apple Watch use (21.4%) exceeded Fitbit Surge (9.5%) and Samsung Gear (9.3%) use in a Chinese sample, and Mannhart et al [28] reported that Apple Watch 6 was selected most frequently (39%) among patients trialing multiple wearables, with brand familiarity often cited as a factor. In our study, Fitbit’s lack of smartphone brand association may have contributed to its lower uptake. Nevertheless, participant satisfaction with all 3 devices was similarly high after use, suggesting that initial perceptions do not necessarily predict longer-term acceptability, underscoring the value of empirically evaluating both user experience and data quality under real-world conditions.

Consistent with our findings, Hofbauer and Rodriguez [26] found no significant difference in usability scores between Fitbit Charge 4, Apple Watch 3, and Polar watch after 2 weeks of use in older adults. Mannhart et al [28] reported that while participants rated the Samsung Galaxy Watch lower than the Apple Watch and Fitbit Sense for comfort, ease of learning, and willingness to use long term, it received the highest score (4.5/5) for ease of putting it on. Similarly, Jia et al [30] found that the Fitbit Surge had the highest overall satisfaction score and ease-of-use scores, whereas the Apple Watch scored higher on design, durability, and additional features. Alshamari and Althobaiti [32] reported greater overall satisfaction with the Samsung Galaxy Watch 5 than the Fitbit Charge 5 after 30 days of use. Collectively, these mixed findings suggest that while overall usability and satisfaction may not differ significantly between devices, specific features, such as design, interface, and ease of use, can influence user experience.

The Fitbit consistently produced the most complete step count, heart rate, and sleep data, whereas the Samsung Galaxy Watch recorded the least across all domains. Hofbauer and Rodriguez [26] similarly found that the Apple Watch had the lowest data completeness, with step count and heart rate data missing for 55.6% and 77.8% of participants, respectively. One-third (9/27) reported removing the device at night, and Apple Watches typically required charging every 1‐2 days compared with 1‐3 times over 2 weeks for Fitbit devices. Wu et al [27] reported greater missing data from the Samsung Galaxy 5 watch compared to an ActiGraph and patch sensor, largely due to daily charging requirements and participants forgetting to rewear the device. Huang et al [40] observed that 6‐8 hours of data were lost every 1‐2 days with Apple Watch (versions 2‐3), primarily during overnight charging. Together, these findings suggest that shorter battery life for the Apple Watch and Samsung Galaxy Watch likely contributed to lower sleep data availability compared to Fitbit, highlighting the importance of battery performance and wearability for maximizing data completeness, particularly for sleep monitoring.

### Limitations

The main limitations of this study are around the quantitative analysis, particularly the difficulty of determining the reasons for differences in data completeness across device groups. It is unclear whether lower data volumes were due to participants not wearing the devices, technical issues with the hardware or proprietary software, or failures in the data flow pipeline, either within or beyond the CONNECT app. Another limitation concerns the inability to reliably assess wear time. Only the Apple Watch provides an explicit indicator of when the device is being worn. While continuous heart rate data could be used as a proxy for wear time, it does not allow exclusion of alternative reasons for missing data, such as technical failures. A further limitation is the lack of direct assessment of data accuracy, which is a critical dimension of wearable data quality [41] and acceptability to users [42], therefore limiting the interpretability of the findings. Although data completeness was used as a proxy for quality, we did not validate the physiological data against gold-standard, research-grade devices (eg, electrocardiogram monitors or accelerometers). When assessing data completeness for objective 2, a valid day was defined as a day with any data recorded, which may overestimate meaningful wear time. This definition was used because step count data from the Samsung Galaxy Watch was only available as daily totals, and we wished to be consistent across the 3 metrics. However, for objective 3, we conducted an additional analysis of heart rate data at the hourly level, which provides a more informative meaningful estimate of wear time.

Additional limitations relate to the sample size and follow-up duration. Of the 105 participants in the cohort, 70 (66.7%) had completed the first WDSQ follow-up assessment at the time of analysis. The sample for the quantitative analyses was further reduced by exclusions relating to the use of personal devices, data file merging issues, and early disengagement, resulting in no recorded wearable data. Participants in the latter category were excluded because all were from the same device group, and their inclusion risked disproportionately biasing the results. For participants with issues downloading and merging data files, it was not clear what was causing these issues and whether they could be resolved, so a decision was made to proceed with the analysis. Although the follow-up period extended to 20 weeks, not all participants contributed data for the full duration. As a result, estimates of long-term engagement and continuity may be biased by attrition or selective drop-out. Additionally, the intended recruitment target of at least 10 participants per site was not met in 2 recruiting sites, each contributing only 7 participants. Given observed demographic differences between sites (Table S1 in Multimedia Appendix 1), under-recruitment at these sites may have contributed to demographic imbalances in the study.

Other limitations relate to the wearable devices themselves, including the way in which they were allocated. Participants were allowed to select their preferred device rather than being randomized, resulting in unequal group sizes and some differences in baseline demographic characteristics, which may have caused differences in device engagement between groups. However, Poisson modeling showed weak evidence of any difference in data completeness attributable to device choice, after adjusting for engagement with the ASM (Table S5 in Multimedia Appendix 1). Participant choice was intentional in this pilot study, as one of the study aims was to assess device preference as part of acceptability to inform likely longer-term engagement in the wider CONNECT participant cohort. While a more rigorous, randomized comparison would be valuable in a different study designed specifically to estimate comparative device performance under balanced baseline conditions, this would not have addressed our objective. In addition, while the number of troubleshooting incidents was reported, we did not formally quantify the associated time burden for research staff or the impact of these technical issues on participant experience. A more detailed analysis of implementation burden would be valuable for informing the scalability of future studies using commercial wearable devices. Additionally, the study focused on commercial-grade wearables, which differ in function, data access, and reliability from research-grade devices commonly used in clinical trials. A further limitation is that findings relate to 3 specific device models evaluated at the current time; given rapid firmware updates and new product releases, performance characteristics may change quickly. Therefore, our findings from this UK psychosis sample may not generalize to other clinical populations, health care systems, or cultural contexts. As such, caution should be exercised in extrapolating these findings to other contexts, particularly within clinical or regulatory settings where more stringent data requirements may apply. However, our use of commercial devices is a strength of the study, given the potential implementation of the CONNECT DRM system in clinical services.

### Conclusions

In this study, evaluating the use of commercially available wearable devices among people with psychosis, we found comparable levels of participant-reported satisfaction across the 3 devices tested. However, while all devices were acceptable from a user perspective, their capacity to produce complete and high-quality data varied substantially. These findings therefore highlight the value of using a nested pilot study to systematically evaluate data integrity when selecting wearables for clinical and research use. This framework represents good practice for future digital health studies using wearable devices. In our case, the Samsung Galaxy Watch was discontinued in the CONNECT study, a decision that reflects a data-driven approach to optimizing passive sensing data collection protocols.

## Supplementary material

10.2196/86049 Multimedia Appendix 1 Supplementary materials with additional tables and figures.

## Abbreviations

ASM: active symptom monitoring
DRM: digital remote monitoring
IMD: Index of Multiple Deprivation
REDCap: Research Electronic Data Capture
WDSQ: Wearable Device Satisfaction Questionnaire
