# Supplementary material for: Evaluating Wearable Devices for Remote Monitoring in Psychosis: Pilot Study Nested Within the CONNECT Cohort Study
Source: JMIR Form Res. 2026 Jul 7;10:e86049. doi: 10.2196/86049 (PMC13340901; doi:10.2196/86049)
Supplement: Multimedia Appendix 1 [file formative-v10-e86049-s001.pdf]

Supplementary Materials

Contents:

Table S1. Breakdown of wearables by smartphone type

Table S2. Baseline demographics of CONNECT pilot participants by recruitment site

Table S3. Summary of step count, sleep and heart rate data for the 3 devices

Figure S1. Percentage of possible hours of heart rate data

Table S4. Estimated length of onboarding sessions by device group

Table S5. Metrics available for each device

Figure S2. Percentage of participants with at least 3 days of data per week

Table S6. Poisson modelling results A

Table S7. Poisson modelling results B

Table S1. Breakdown of wearable device by phone type

Wearable at Onboarding	CONNECT Phone - Android	Own phone - Android	Own phone - iOS
Apple Watch	0 (0.0%)	0 (0.0%)	28 (80.0%)
Samsung Galaxy	39 (67.2%)	8 (66.7%)	0 (0.0%)
Fitbit	17 (29.3%)	4 (33.3%)	3 (8.6%)
No wearable	2 (3.4%)	0 (0.0%)	4 (11.4%)

Table S2. Baseline demographics of CONNECT pilot participants by recruitment site.

Demographic Characteristic	All, n = 105	Cardiff, n = 17	Edinburgh, n = 7	Glasgow, n = 7	KCL, n = 29	Manchester, n = 30	Sussex, n = 15
**Age, median (IQR)**	33.0 (24.0, 45.0)	22.0 (21.0, 24.0)	44.0 (29.5, 58.0)	46.0 (44.0, 56.5)	32.0 (27.0, 43.0)	31.0 (25.0, 38.0)	41.0 (31.0, 47.0)
**Age Group, n (%)**							
16-20	7 (6.7%)	4 (23.5%)	1 (14.3%)	0 (0.0%)	0 (0.0%)	2 (6.7%)	0 (0.0%)
21-30	38 (36.2%)	9 (52.9%)	1 (14.3%)	1 (14.3%)	11 (37.9%)	12 (40.0%)	4 (26.7%)
31-40	23 (21.9%)	1 (5.9%)	1 (14.3%)	0 (0.0%)	8 (27.6%)	10 (33.3%)	3 (20.0%)
41-50	18 (17.1%)	2 (11.8%)	1 (14.3%)	4 (57.1%)	3 (10.3%)	3 (10.0%)	5 (33.3%)
51-60	13 (12.4%)	1 (5.9%)	1 (14.3%)	0 (0.0%)	7 (24.1%)	2 (6.7%)	2 (13.3%)
61-70	6 (5.7%)	0 (0.0%)	2 (28.6%)	2 (28.6%)	0 (0.0%)	1 (3.3%)	1 (6.7%)
70+	0 (0.0%)	0 (0.0%)	0 (0.0%)	0 (0.0%)	0 (0.0%)	0 (0.0%)	0 (0.0%)
**Ethnic Group, n (%)**							
Asian/ Asian British	13 (12.4%)	3 (17.6%)	0 (0.0%)	1 (14.3%)	4 (13.8%)	5 (16.7%)	0 (0.0%)
Black/ Black British/ Caribbean/ African	22 (21.0%)	2 (11.8%)	0 (0.0%)	0 (0.0%)	15 (51.7%)	5 (16.7%)	0 (0.0%)
Mixed/ multiple ethnic groups	10 (9.5%)	1 (5.9%)	1 (14.3%)	0 (0.0%)	2 (6.9%)	5 (16.7%)	1 (6.7%)
White	56 (53.3%)	9 (52.9%)	6 (85.7%)	6 (85.7%)	7 (24.1%)	15 (50.0%)	13 (86.7%)
Other ethnic group	4 (3.8%)	3 (16.7%)	0 (0.0%)	0 (0.0%)	1 (3.4%)	0 (0.0%)	1 (6.7%)
**Sex, n (%)**							
Female	46 (43.8%)	7 (41.2%)	2 (28.6%)	2 (28.6%)	11 (37.9%)	19 (63.3%)	5 (33.3%)
Male	59 (56.2%)	10 (58.8%)	5 (71.4%)	5 (71.4%)	18 (62.1%)	11 (36.7%)	10 (66.7%)
**Employment Status, n (%)**							
Self employed	1 (1.0%)	0 (0.0%)	1 (14.3%)	0 (0.0%)	0 (0.0%)	0 (0.0%)	0 (0.0%)
Employed	16 (15.2%)	2 (11.8%)	2 (28.6%)	0 (0.0%)	5 (17.2%)	5 (16.7%)	2 (13.3%)
Out of work and looking for work	20 (19.0%)	4 (23.5%)	0 (0.0%)	1 (14.3%)	10 (34.5%)	5 (16.7%)	0 (0.0%)
Out of work and not looking	20 (19.0%)	0 (0.0%)	1 (14.3%)	0 (0.0%)	8 (27.6%)	9 (30.0%)	2 (13.3%)
Unable to work	25 (23.8%)	2 (11.8%)	3 (42.9%)	4 (57.1%)	0 (0.0%)	9 (30.0%)	7 (46.7%)
Voluntary work	7 (6.7%)	3 (17.6%)	0 (0.0%)	0 (0.0%)	3 (10.3%)	0 (0.0%)	1 (6.7%)
Student	13 (12.4%)	7 (41.2%)	0 (0.0%)	0 (0.0%)	2 (6.9%)	1 (3.3%)	3 (20.0%)
Retired	4 (3.8%)	0 (0.0%)	1 (14.3%)	2 (28.6%)	0 (0.0%)	1 (3.3%)	0 (0.0%)

Other	1 (1.0%)	0 (0.0%)	0 (0.0%)	0 (0.0%)	1 (3.4%)	0 (0.0%)	0 (0.0%)
**Deprivation Quintile, n (%)**							
1 (most deprived)	41 (39.0%)	7 (38.9%)	1 (14.3%)	4 (57.1%)	8 (27.6%)	19 (63.3%)	2 (13.3%)
2	26 (24.8%)	1 (5.6%)	2 (28.6%)	1 (14.3%)	15 (51.7%)	4 (13.3%)	3 (20.0%)
3	18 (17.1%)	5 (27.8%)	0 (0.0%)	0 (0.0%)	6 (20.7%)	4 (13.3%)	4 (26.7%)
4	13 (12.4%)	3 (16.7%)	2 (28.6%)	1 (14.3%)	0 (0.0%)	2 (6.7%)	5 (33.3%)
5 (least deprived)	6 (5.7%)	2 (11.1%)	1 (14.3%)	1 (14.3%)	0 (0.0%)	1 (3.3%)	1 (6.7%)
Missing	1 (1.0%)	0 (0.0%)	1 (14.3%)	0 (0.0%)	0 (0.0%)	0 (0.0%)	0 (0.0%)
**In Receipt of Welfare Benefits, n (%)**							
Yes	87 (82.9%)	13 (76.5%)	4 (57.1%)	6 (85.7%)	25 (86.2%)	25 (83.3%)	14 (93.3%)
No	16 (15.2%)	4 (23.5%)	2 (28.6%)	0 (0.0%)	4 (13.8%)	5 (16.7%)	1 (6.7%)
Prefer not to say	1 (1.0%)	0 (0.0%)	0 (0.0%)	1 (14.3%)	0 (0.0%)	0 (0.0%)	0 (0.0%)
Missing	1 (1.0%)	0 (0.0%)	1 (14.3%)	0 (0.0%)	0 (0.0%)	0 (0.0%)	0 (0.0%)

Table S3. Summary of step count, sleep and heart rate data for the 3 devices. There was no significant difference between the number of possible days of data, p = 0.606. For the median number of HR hours – The difference between the Fitbit and Samsung Galaxy groups was statistically significant (p = 0.007), while differences between Fitbit and Apple watch (p = 0.262) and between Apple watch and Samsung Galaxy (p = 0.168) were not statistically significant.

		**Apple watch (n = 27)**	**Fitbit (n = 20)**	**Samsung Galaxy (n = 40)**
**Number of possible days of data per person, median (IQR)**		119.0 (101.0, 132.5)	131.5 (104.0, 148.0)	128.5 (103.0, 162.8)
**Step Count**	No. of participants	27 (100%)	20 (100%)	40 (100%)
	Median no. of days (IQR)	72.0 (35.5, 89.0)	88.5 (27.8, 109.8)	44.5 (17.8, 86.0)
	Median % of possible days (IQR)	64.3 (30.0, 76.8)	74.8 (32.1, 96.9)	37.5 (15.4, 60.7)
**Heart Rate**	No. of participants	27 (100%)	20 (100%)	38 (95.0%)
	Median no. of days (IQR)	61.0 (27.0, 100.5)	87.5 (31.0, 125.5)	38.0 (11.8, 57.3)
	Median % of possible days (IQR)	49.3 (21.5, 86.0)	80.1 (26.7, 95.0)	31.2 (8.5, 46.0)
	Median no. of hours (IQR)	1,002.0 (288.0, 1,470.0)	1,440.5 (452.3, 2,386.0)	601.5 (129.0, 845.5)
	Median % of possible hours (IQR)	28.8 (10.5, 57.6)	53.0 (21.3, 92.4)	15.8 (3.4, 30.1)
**Sleep**	No. of participants	19 (70.4%)	19 (95.0%)	32 (80.0%)
	Median no. of days (IQR)	31.0 (0.0, 81.0)	41.0 (13.8, 108.0)	5.5 (2.0, 23.0)
	Median % of possible days (IQR)	23.6 (0.0, 56.1)	49.4 (12.5, 87.3)	4.8 (1.9, 17.6)

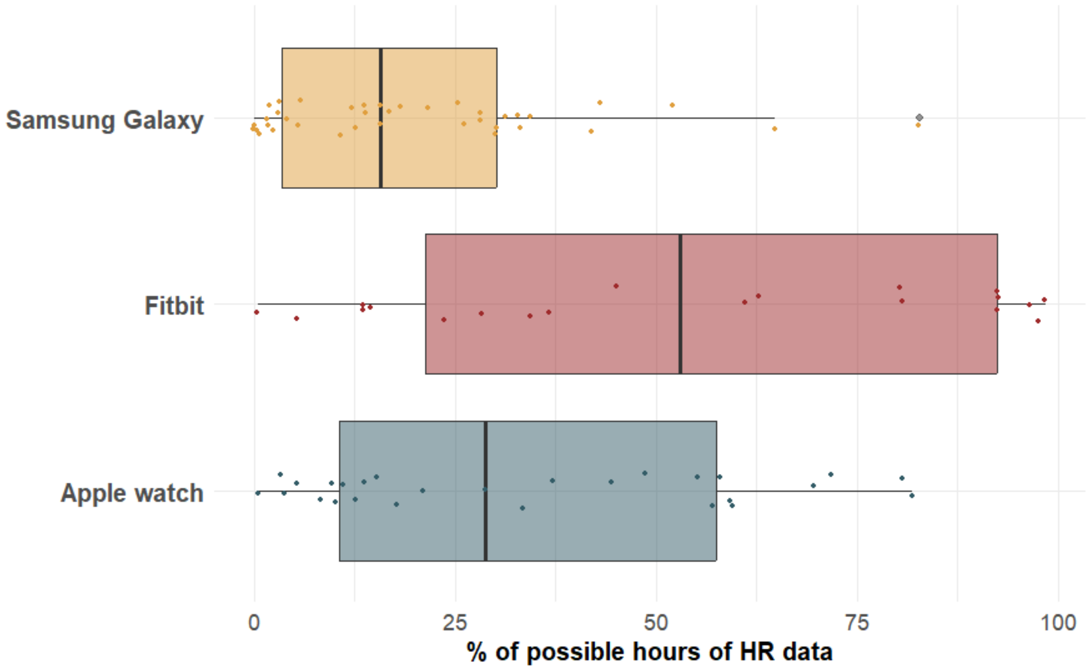

Figure S1. Percentage of possible hours of heart rate data. The number of hours where at least 1 heart rate measurement was recorded was calculated for each participant as a % of the number of total possible hours of data, assuming 24-hour wear for all days of follow-up. The Fitbit group had a significantly higher % of heart rate data than the Galaxy group ($p = 0.0013$) but not the Apple watch group ($p = 0.1304$). The difference between the Galaxy and Apple watch groups was also not significant ($p = 0.1098$).

Table S4. Estimated length of onboarding sessions by device group.

		Estimated Onboarding Time (mins)		
**by Wearable**	**N of Participants**	**Range**	**Median (IQR)**	**N over 2 hours**
Apple Watch	21	10, 150	60 (60, 120)	1 (5.0%)
Samsung Galaxy	17	30, 360	60 (30, 180)	5 (29.4%)
Fitbit	15	45, 360	90 (83, 120)	3 (20.0%)

Table S5. Metrics available for each device

Data type	Apple watch	Fitbit	Samsung Galaxy
Step count	Yes (every few seconds)	Yes (minute total)	Yes (daily total)
Heart rate	Yes (every 5-10 seconds)	Yes (every 5-10 seconds)	Yes (every 5-10 seconds)
Sleep	Yes	Yes	Yes
Activities/exercise log	Yes	Yes	Yes
Heart rate variability	Yes	Yes	No
Respiration rate	Yes	Yes	No
Resting heart rate	Yes	No	No
Calorie expenditure	No	Yes	No
Skin temperature	No	Yes	No
Ambient pressure	Yes	No	No
Ambient light	Yes	No	No
Accelerometer	Yes	No	No

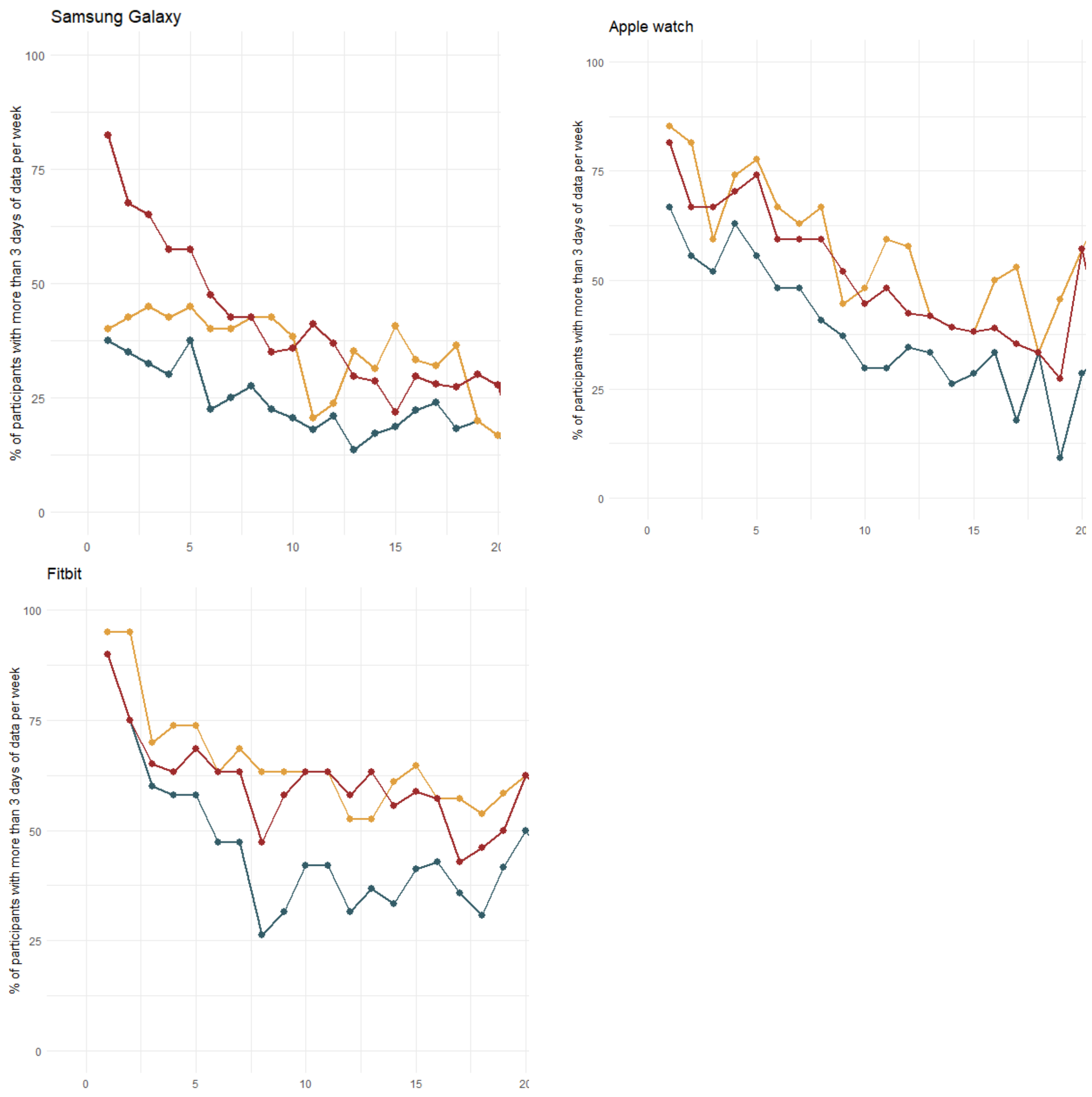

Figure S2. **Percentage of participants with at least 3 days of data per week**, for Active Symptom Monitoring (ASM) responses (red line), passive heart rate data (yellow line) and both passive and ASM data on the same day (blue line

Table S6. Poisson modelling results A. Models not including baseline demographics.

	**model i) - unadjusted**			**model ii) - offset by no. of ASM days**		
	IRR	95% CI	p-value	IRR	95% CI	p-value
**Wearable device**						
**Apple watch (ref)**	—	—		—	—	
**Fitbit**	1.26	0.88, 1.80	0.20	1.02	0.66, 1.55	>0.9
**Samsung Galaxy**	0.59	0.41, 0.84	0.005	0.71	0.46, 1.08	0.11

We developed two Poisson models utilising ASM data to adjust for differences between wearable subgroups:

i) unadjusted model with the number of days of passive heart rate data as the outcome and wearable device as the only variable. To account for the participants being in the pilot for different lengths of time the log of the number of possible days (i.e., total number of days on study for each individual) was included as an offset

ii) model with the number of days of passive data as the outcome and wearable device as the only variable, with the log of the number of completed ASM days as an offset in the model.

There is no indication of a difference between data completeness attributable to Fitbit versus Apple watch, and very weak evidence that lower data completeness is attributable to the Samsung Galaxy

Table S7. Poisson modelling results B. Models adjusting for baseline demographics.

	Model i)			Model ii)		
	**IRR**	**95% CI**	**p-value**	**IRR**	**95% CI**	**p-value**
**Wearable device**						
Apple watch	—	—		—	—	
Fitbit	1.05	0.64, 1.73	0.9	1.02	0.66, 1.58	>0.9
Samsung Galaxy	0.54	0.34, 0.86	0.012	0.75	0.51, 1.12	0.2
**Age**	1.01	0.99, 1.02	0.3	0.99	0.98, 1.00	0.14
**Ethnic group**						
White	—	—		—	—	
Asian/ Asian British	1	0.62, 1.56	>0.9	1.26	0.84, 1.85	0.3
Black/ Black British/ Caribbean/ African	0.61	0.35, 1.01	0.066	0.83	0.52, 1.30	0.4
Mixed/ multiple ethnic groups	0.9	0.41, 1.76	0.8	1.1	0.56, 1.99	0.8
Other ethnic group	2.03	0.69, 5.81	0.2	2.94	1.07, 7.90	0.038
**Sex**						
Female	—	—		—	—	
Male	0.89	0.61, 1.28	0.5	1.38	1.01, 1.90	0.048
**Employment status**						
employed/self-employed	—	—		—	—	
Out of work or unable to work	1.02	0.64, 1.66	>0.9	0.79	0.53, 1.20	0.3
Retired	1.02	0.36, 2.68	>0.9	0.9	0.38, 2.05	0.8
Student	0.85	0.41, 1.74	0.7	0.67	0.35, 1.28	0.2
Voluntary work	0.87	0.35, 2.03	0.8	0.98	0.44, 2.05	>0.9
**In receipt of welfare benefits**						
No	—	—		—	—	
Yes	0.91	0.55, 1.53	0.7	0.86	0.56, 1.33	0.5
**Deprivation quintile**						
1	—	—		—	—	
2	0.94	0.58, 1.50	0.8	1.09	0.72, 1.63	0.7
3	0.63	0.34, 1.12	0.14	1.23	0.75, 1.96	0.4
4	0.95	0.54, 1.64	0.9	1.17	0.71, 1.91	0.5
5	0.86	0.41, 1.74	0.7	0.56	0.29, 1.04	0.082

Model i) includes an offset for the log number of possible days of data, to account for participants having different lengths of follow-up. Model ii) includes an offset for the log number of completed ASM encounters, to account for different levels of engagement. The models show that age, ethnic group, employment status, receipt of welfare benefit and deprivation quintile were not significantly associated with passive data completeness. In model ii) being male was significantly associated with higher passive data completeness.
